# Access and use of human tissues from the developing world: ethical challenges and a way forward using a tissue trust

**DOI:** 10.1186/1472-6939-12-2

**Published:** 2011-01-25

**Authors:** Claudia I Emerson, Peter A Singer, Ross EG Upshur

**Affiliations:** 1 McLaughlin-Rotman Centre for Global Health, University Health Network & University of Toronto, 101 College Street, Suite 406, Toronto, Ontario, M5G 1L7, Canada; 2 University of Toronto Joint Centre for Bioethics, 155 College Street, Suite 754, Health Sciences Bldg, Toronto, Ontario, M5T 1P8, Canada; 3 Department of Family and Community Medicine, University of Toronto, Canada

## Abstract

**Background:**

Scientists engaged in global health research are increasingly faced with barriers to access and use of human tissues from the developing world communities where much of their research is targeted. In part, the problem can be traced to distrust of researchers from affluent countries, given the history of 'scientific-imperialism' and 'biocolonialism' reflected in past well publicized cases of exploitation of research participants from low to middle income countries.

**Discussion:**

To a considerable extent, the failure to adequately engage host communities, the opacity of informed consent, and the lack of fair benefit-sharing have played a significant role in eroding trust. These ethical considerations are central to biomedical research in low to middle income countries and failure to attend to them can inadvertently contribute to exploitation and erode trust. A 'tissue trust' may be a plausible means for enabling access to human tissues for research in a manner that is responsive to the ethical challenges considered.

**Summary:**

Preventing exploitation and restoring trust while simultaneously promoting global health research calls for innovative approaches to human tissues research. A tissue trust can reduce the risk of exploitation and promote host capacity as a key benefit.

## Background

Access and use of human tissues from the developing world is a significant challenge for scientists engaged in global health research. Given the troubled history of 'scientific-imperialism' and 'biocolonialism' in cases of exploitation in research involving vulnerable populations [1-6], many communities and governments in low-to-middle income countries (LMICs) are understandably reluctant to trust foreign researchers and permit access to human tissues. The refusal of the Indonesian government to share its tissue samples of the H5N1 virus with the international community is perhaps the most acute recent example [7]. The biomedical literature is filled with numerous examples of researchers who were welcomed into a community, conducted their research, and then left without returning any meaningful benefit to the studied population. In some cases, researchers appropriated human tissues from the host community without obtaining proper informed consent. Consider:

"[B]lood samples were extracted from some members of the Hagahai, a small group of hunter-gatherers living in an inaccessible mountain range in Papua New Guinea. The researcher involved told the group that she wanted to see a 'binitang' -- an insect -- in their blood. Analysis of these blood samples revealed existence of antibodies to a variant of the HTLV-I leukaemia virus. This was used to produce an immortal cell line, which was the basis for a patent application..." [3]

"[T]he 400 blood samples from members of Arizona's Havasupai Tribe, which are at the centre of a lawsuit against Arizona University, and which allegedly formed the basis for no less than 23 scholarly papers, articles and dissertations". The lawsuit claims the Tribe's genetic information was used for research purposes not contemplated in the consent process [4].

"[A]s the Karitiana Indians remember it, the first researchers to draw their blood came here in the late 1970s...In 1996, another team visited, promising medicine if the Karitiana would just give more blood, so they dutifully lined up again. But that promise was never fulfilled..." [2]

Central to biomedical research in low to middle income countries (LMICs) are three inter-related challenges: informed consent, community engagement, and benefit sharing. Failure to adequately address these challenges can lead to exploitation and the erosion of trust. While these challenges have been addressed individually in research guidelines, specific difficulties encountered by researchers with respect to access and use of human tissue samples in LMICs, and tighter country-level restrictions in this regard [8,9] suggest a more holistic solution is needed. In particular, there is a need to develop approaches to human tissues research in LMICs that are more responsive to exploitation, engaging participants as legitimate stakeholders and not only as subjects of research.

In this article, we briefly examine these three challenges and present a proposal for a tissue trust, a means to address the challenges and the issue of exploitation with respect to access and use of human tissues for research in developing countries.

## Discussion

### Three Inter-related Ethical challenges: Informed Consent, Community Engagement, Benefit Sharing

#### Informed consent

Informed consent remains one of the most vexing challenges for research involving participants from LMICs [10]. Questions arise about the validity of consent -- whether participants were coerced, whether they understood the risks and benefits of the research, whether they were fully informed about how their data and tissues would be used, and whether the consent process was culturally appropriate. Autogen, the Australian company that was refused a proposal to collect tissue samples from Tongans to study diabetes, serves as an example of where the informed consent process was deemed culturally inappropriate because community values were overlooked by favoring an individualistic approach [5,11].

Another example of the challenge of informed consent is where tissues are subjected to uses or handling that were not made explicit, but could be implied or inferred from what is agreed upon in the consent. For example, a participant agrees that their blood sample will be subjected to analysis involving a particular method, but the method is locally unavailable and it is necessary to export the tissue out of the country --something which the participant objects to, but has not explicitly articulated. This highlights a key problem of informed consent: that it is opaque and can be significantly limited in practice [10]. It also highlights the importance of engaging with the host community so that proper determinations regarding values and preferences can be made, and proper disclosure with respect to handling and uses of tissues is accounted for, including the possibility of unknown future research uses and commercialization.

#### Community engagement

Community engagement is recognized by international research ethics guidelines which have declared it an ethical requirement for research involving human participants [12,13]. In the context of research involving human tissues from LMICs, community engagement is essential for gaining an understanding of the special significance tissues may hold, and thus for negotiating culturally acceptable means of access and use. Moreover, community engagement is crucial for discovering preferences with respect to the informed consent process and understanding what constitutes fair distribution of research benefits from the perspective of participants [1].

The failure to effectively engage communities can be seen as a discounting of their values. As a result, participants may come to view research as the imposition of a foreign agenda, eventually rejecting it. "Trials in developing countries have been halted or suspended for a variety of reasons including lack of consensus on ethical issues, lack of appropriate care and treatment of participants, and lack of adequate community consultation [14]". The tenofovir trials are a stark example of where community engagement was seen to have failed.

#### Benefit-sharing

Benefit-sharing initially emerged as a mechanism to restore equilibrium to the asymmetry between researcher and participant when it became increasingly visible that bioscience researchers, companies and universities were accruing profits as a result of research [15]. 'Giving something back' to participants is intended to mitigate exploitation, and benefit-sharing is now generally considered a specific principle of research governance consistent with social justice. In developing world contexts, benefit-sharing is considered a moral imperative given the acute risk for exploitation of the poor and sick and the desperate lack of resources.

In the past, the benefits for research participants in LMICs have not been adequately or justly realized. Some benefit-sharing agreements have been clearly inappropriate, e.g. DNA in exchange for toothbrushes [11]; while other agreements have aimed to be fair and equitable and still judged to be lacking from the perspective of the community, e.g. Autogen and the Tongan people [1]. The 2002 Bonn Guidelines offer a list of possible benefits, both monetary (e.g. access fees, up front payment, joint ownership of relevant IP rights) and non-monetary (sharing of research and development results, collaboration, development programs to build local capacity) [16], but questions of what counts as an appropriate return for the research context, or who is the ultimate beneficiary, still need to be negotiated between researcher and participant communities. Empirical research in Africa has shown, that among other reasons, lack of study benefits is a deterrent to participation in research [17].

### A Tissue Trust

In response to these ethical challenges, we propose a 'tissue trust' as a means of facilitating research involving human tissues. By reducing exploitation through the ratcheting up of local scientific capacity, the tissue trust is responsive to the ethical challenges outlined above. First, enabling tissue donors and community members to participate in research governance ensures that the consent of donors for access and use of tissues is respected and reflective of community expectations. Second, a well devised community engagement process that must necessarily precede any attempts to implement a tissue trust will account for the values and needs of the host community and provide guidance for the governance of the trust. Lastly, a tissue trust is capable of returning long term benefits to the source community that extend beyond the fruits of research itself, such as the improvement of healthcare facilities and the training of local personnel which contributes to development.

The tissue trust is intended to serve the interests of the common good and not just those that make contributions. Thorsteinsdóttir et al have argued that in principle genomics is a global public good, and what is limiting this in practice is access goods [18] - this would be provided by a tissue trust. This notion of the public good finds support in the view that the fruits of genetic research should be shared by all of humanity, as espoused by the Human Genome Organisation (HUGO) [19]. As well, the view of tissue resources as a common good is preferable to conceptualizing tissues as a proprietary good, which can elicit conflicts of ownership and risk of commodification.

The idea of a tissue trust for managing tissue collections assembled for research is inspired by Winickoff and Winickoff's proposal for a charitable trust model for genomic biobanks. In the Winickoffs' model, tissues are held in trust for the donors by a trustee who oversees uses in accordance with the wishes of the beneficiaries of the trust; in this case the general public [20]. The tissue trust shares with the charitable trust common features, namely having an independent third party administer the trust on behalf of contributors for the benefit of the wider community. The novel and distinctive feature of the tissue trust is that it is specifically suited for the developing world context.

#### Mission and vision

The aim of the tissue trust is to build scientific capacity in the developing world, to facilitate local research and reduce exploitation by eliminating the need for exportation of tissues. To this end, the trust is designed with mechanisms for promoting host capacity: it requires justification for the exportation of tissues and exacts a fee from users submitting such requests. Over time, these fees accumulate and are used for local investment in scientific infrastructure, which returns benefits to the host community over a sustained period of time that are more fruitful than short-lived upfront payments to donors or back-end payments contingent on successful patents or commercialization. As local scientific capacity builds up, the need to export tissues from the region is gradually reduced and eventually eliminated. This gradual 'ratcheting up' in capacity is consistent with the vision of international research ethics Benatar and Singer proposed almost a decade ago, aiming for the improvement of standards rather than the setting of unrealistic goals [21].

#### Governance

Governance of the trust is through a board of trustees that acts as a fiduciary on behalf of the source community, ensuring their interests are protected. The advantage of having a fiduciary administer the trust is that it minimizes the conflict of ownership which can arise between tissue donors and the repositories they contribute to. With a trust, no ownership rights are relinquished; rather ownership interests are transferred to the trustee to manage according to the donor's wishes. The wishes of the source community are made explicit through an effective community engagement process, which naturally must precede the structuring of the tissue trust. Access and use of tissues can remain consistent with the expectations of the donors through their participation in the governance of the trust; for example, through membership on the board, the research ethics committee (REC) or a donor committee with veto power over particular projects [20]. The key element is to engage the community upfront and sustain that engagement through meaningful participation and buy-in into the trust.

#### Organizational structure

The tissue trust is a model that can be applied on a project, institutional, national, or regional basis. The trust is structured as an independent non-profit entity with its own board of trustees comprised of stakeholders that include at least one elected member from the donor community.

An obvious location for the physical infrastructure of a tissue trust is at an institution with some infrastructure already in place, such as a hospital. The Winickoffs outline three advantages for selecting an established medical institution: the unique relationships these institutions have with tissue and blood donors; the means to attract public and private funds; and the capacity to install good governance structures that can motivate potential tissue donors [20]. Concerns regarding the dominance of the host institution in managing the tissue resource are eliminated by having a separate non-profit organizational structure and governance that spans the interests of relevant stakeholders. A data steward facilitates access and distribution of the tissues in accordance with direction from the board of trustees. Additionally, the trust has its own REC to review the scientific and ethical merits of projects proposing access.

#### Business models and funding

Donors contribute tissues, while users proposing to export tissues contribute funds. According to the Winickoffs, biobanks can attract investment when public benefit, rather than profit, serves as the organizing principle [20]. To this extent, initial funding for infrastructure for the tissue trust could be supplied by institutions having a stake in public health, e.g. health departments, national research funding bodies, and foundations. Thereafter, access fees for tissues requiring exportation would be applied and those levies used for scientific capacity building. This raises the question of whether sufficient funds will be generated through such a mechanism to make a meaningful contribution to building capacity; at present this is unknown. A limitation of the tissue trust is that it is a concept that remains to be tested. Moreover, further empirical research is required to ascertain the volume of tissues exported from the developing world, but there is some indication that this may be substantial. It has been reported that Uganda is losing millions of dollars due to exportation of human biological samples [1].

As well, partnerships with the private sector should not be discounted as a source of funding. In their account of the charitable trust, the Winickoffs urge trustees to apply funding models that seek research partnerships instead of tissue buyers. They offer the example of one non-profit tissue bank that has successfully implemented a partnership with the private sector:

"PXE international --a rare-disease group that has established a nonprofit blood and tissue bank --has generated funding by negotiating intellectual-property arrangements with commercial researchers. "[20]

As a concept, the tissue trust may be seen as an ideal model. However, the underlying principles of promoting southern governance and increasing research capacity in LMICs are broadly applicable to projects in the developing world, and can be adopted even where formal structural organization or business models are lacking.

## Summary

To facilitate the use of human tissues for global health research it is important to understand the ethical challenges that presently stand in the way of access. In the past, informed consent has not always been properly obtained, communities were not sufficiently engaged, and meaningful benefits were not returned to the source community. These practices have contributed to the exploitation of participants and served to undermine trust in research. To respond to concerns of exploitation that can arise in the access and use of human tissues from the developing world, we propose a tissue trust. The trust is a fund to which contributions are held and used for the development of scientific infrastructure in the developing world. In effect, the trust becomes an investment tool used to build up local capacity, so that eventually research may be conducted locally and exportation of tissues can be reduced.

## Competing interests

The authors declare that they have no competing interests.

## Authors' contributions

All authors contributed to the conceptual development of the paper and manuscript revisions. CIE drafted the manuscript. All authors read and approved the final manuscript.

## Pre-publication history

The pre-publication history for this paper can be accessed here:

http://www.biomedcentral.com/1472-6939/12/2/prepub
